# Impaired thyroid hormone sensitivity is associated with the degrees of fatty infiltration in metabolic dysfunction associated steatotic liver disease

**DOI:** 10.3389/fendo.2025.1646790

**Published:** 2025-09-19

**Authors:** Ying Li, Fang Wang

**Affiliations:** ^1^ Department of Endocrinology and Metabolism, The First People’s Hospital of Hefei, Hefei, Anhui, China; ^2^ Department of The Health Management Center, The First Affiliated Hospital of University of Science and Technology of China (USTC): Anhui Provincial Hospital (South District), Hefei, Anhui, China

**Keywords:** controlled attenuation parameters, liver fatty infiltration, metabolic dysfunction associated steatotic liver disease, thyroid hormone sensitivity, thyroid feedback quantile-based index

## Abstract

**Background:**

As a chronic disease, MASLD seriously endangers human health and has a complex pathogenesis. Thyroid hormones (THs) play significant roles in this process. We aimed to analyze the correlation between TH sensitivity and the degrees of fatty infiltration in MASLD.

**Methods:**

We conducted a retrospective study on a sample of 13,144 individuals who underwent physical examinations. Thyroid function, liver and kidney function, blood lipids, and glucose were measured using chemiluminescence methods. TH sensitivity indexes, including free triiodothyronine to free thyroxine ratio (FT3/FT4), thyroid feedback quantile-based index (TFQI), thyroid-stimulating hormone index (TSHI), and thyrotropin thyroxine resistance index (TT4RI), were calculated. The degree of liver fatty infiltration [controlled attenuation parameter (CAP)] was determined by liver shear wave quantification ultrasonography. We then conducted statistical analyses of the above data.

**Results:**

FT3/FT4, TFQI, TSHI, and TT4RI showed significantly increasing trends with the rise of CAP levels (p < 0.001). In males, high CAP levels of CAP were negatively correlated with FT3/FT4 (β [95% CI]: −0.005 [−0.008, −0.002]; p = 0.0004) but positively correlated with TSHI (β [95% CI]: 0.019 [0.002, 0.036]; p = 0.0248) and TFQI (β [95% CI]: 0.015 [0.003, 0.027]; p = 0.01371). In the BMI <28 kg/m² group, low CAP levels of CAP were positively correlated with FT3/FT4 (β [95% CI]: 0.002 [0.002, 0.003]; p < 0.00001), while high CAP levels of CAP were positively correlated with TFQI (β [95% CI]: 0.016 [0.000, 0.031]; p = 0.04805).

**Conclusions:**

TH sensitivity is significantly impaired in MASLD. This phenomenon is more pronounced in males and in individuals with BMI <28 kg/m².

## 1 Introduction

The definition of metabolic dysfunction–associated steatotic liver disease (MASLD) was proposed in the international joint guidelines on 24 June 24, 2023 as a reclassified definition (new nomenclature of nonalcoholic fatty liver disease [NAFLD]). MASLD refers to the presence of hepatic steatosis [imaging tests suggesting fatty liver, or liver tissue pathological biopsy (the gold standard) indicating fatty change in more than 5% of hepatocytes], accompanied by one or more cardiovascular metabolic risk factors, while excluding fatty liver disease caused by long-term excessive consumption of alcohol consumption (1). MASLD is regarded as the manifestation of metabolic syndrome in the liver, and its incidence has increased along with the rising prevalence of obesity and type 2 diabetes (2). MASLD is not only the main cause of liver cirrhosis, hepatocellular carcinoma, liver transplantation, and liver-related deaths, but also increases the mortality of cardiogenic diseases (3), the risk of stroke and cerebrovascular diseases, and even increased the risk of developing cancers such as breast cancer and colorectal cancers (4).

Thyroid hormones (THs) regulate the functions of digestive system function and heat production in the body, and modulate lipid metabolism in the liver (5). Under physiological conditions, due to the negative feedback of the hypothalamic–pituitary–thyroid axis, thyroid-stimulating hormone (TSH) shows a negative correlation with free thyroxine (FT4) (6). However, we observed high level of TSH levels may coexist with high level of FT4 levels, rather than the typical presentation of TH resistance (7). Therefore, several scholars have proposed the concept of central TH sensitivity, including the thyroid feedback quantile-based index (TFQI) (7), thyroid-stimulating hormone index (TSHI) (8), and thyrotropin thyroxine resistance index (TT4RI) (9), which have been associated with various metabolic diseases, including hyperuricemia and hyperhomocysteinemia (10, 11). The ratio of free triiodothyronine to free thyroxine (FT3/FT4) is used to evaluate the conversion rate of T4 to T3, reflecting the TH sensitivity of peripheral tissues. This ratio has been shown to be closely related to high lipid levels of lipid (12), and to an increased risk of prediabetes (13) and MASLD (14).

Since THs play crucial roles in lipid metabolism in the liver, it is hypothesized that the lipid deposition in MASLD may lead to cellular dysfunction of cells, abnormal expression of TH receptors, and the impaired sensitivity to THs. We therefore conducted a retrospective analysis of the data from a physical examination population, selecting individuals with normal thyroid function who had undergone liver shear wave quantification ultrasonography. We recorded controlled attenuation parameter (CAP) values of CAP and performed fatty infiltration grading of MASLD. Finally, we analyzed the correlation between the degree of liver fatty infiltration and TH sensitivity indices (FT3/FT4, TT4RI, TSHI, and TFQI) to infer the impact of liver fatty infiltration on TH sensitivity.

## 2 Subjects and methods

### 2.1 Studied subjects

**Figure 1:**
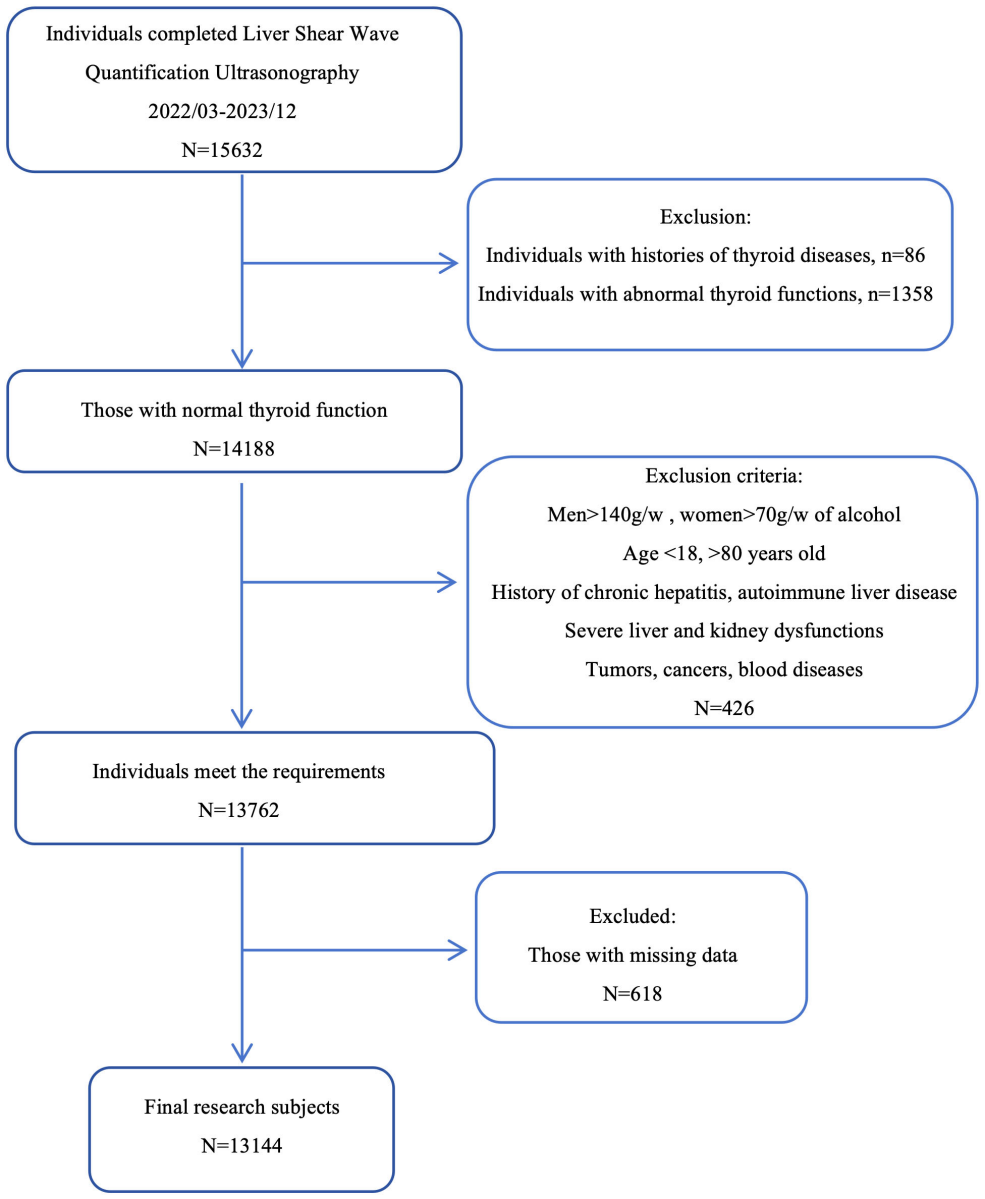
Clinical screening process for the research subjects.

We selected 15,632 individuals who underwent liver shear wave quantification ultrasonography (Mindray, Shenzhen, China) at the Physical Examination Center of the First Affiliated Hospital of the University of Science and Technology of China from March 2022 to December 2023. The study population was drawn from the same city and consisted mainly consisting of individuals engaged in light physical labor, along with some freelancers. We employed the complete case analysis method. For individuals with missing data, the information was excluded. Based on the exclusion and inclusion and exclusion criteria as the evaluation conditions, 13,144 individuals were finally selected as the study subjects (**Figure 1**).

### 2.2 Ethical approval

This study was approved by the Ethics Committee of the First Affiliated Hospital of the University of Science and Technology of China in accordance with the Declaration of Helsinki. Because this was a retrospective study, the Ethics Committee waived the requirement for written informed consent. Approval number: 2024-RE-202.

### 2.3 Inclusion criteria and exclusion criteria

Inclusion criteria: Age between 18 to 80 years old (inclusive). Exclusion criteria: ① History of thyroid disorders, including hyperthyroidism, hypothyroidism, Hashimoto’s thyroiditis, and other thyroid diseases; ② Individuals with abnormal thyroid function, deviating from the normal range of FT3 (2.77 - 6.31 pmol/L), FT4 (10.44 - 24.38 pmol/L), and TSH (0.38 - 4.34 mIU/L). Because some cases of simple obesity may develop subclinical hypothyroidism, the screening range for TSH was extended to 8 mIU/L; ③ History of excessive alcohol consumption, defined as men consuming more than 140g per week and women consuming more than 70g per week; ④ History of chronic viral hepatitis, autoimmune liver disease, liver cirrhosis or other chronic liver diseases; ⑤ Severe liver or kidney dysfunction; ⑥ Individuals with histories of malignant tumors, cancer or malignant hematological diseases; ⑦ Pregnant or lactating women.

### 2.4 Clinical information

We collected basic clinical information, including sex, age, and history of chronic diseases such as diabetes, hypertension, and hyperlipidemia. Height, weight, systolic blood pressure, and diastolic blood pressure were measured in a resting state. Fasting blood samples were drawn to measure the following indicators: fasting blood glucose (FBG, 3.9–6.1 mmol/L), glycated hemoglobin A1c (HbA1c, 4.0%–6.4%), triglycerides (TG, 0–1.7 mmol/L), total cholesterol (T-CHO, 0–5.18 mmol/L), low-density lipoprotein cholesterol (LDL-C, 0–3.37 mmol/L), high-density lipoprotein cholesterol (HDL-C, 1.04–1.55 mmol/L), alanine aminotransferase (ALT, 9–50 IU/L), aspartate aminotransferase (AST, 15–40 IU/L), γ-glutamyl transpeptidase (GGT, 10–60 IU/L), total bilirubin (TBIL, 3.4–21.0 μmol/L), albumin (ALB, 40-55g/L), serum creatinine (SCr, 57–111 μmol/L), serum uric acid (SUA, 208–428 μmol/L), FT3 (2.77–6.31 pmol/L), FT4 (10.44–24.38 pmol/L), and TSH (0.38–4.34 mIU/L).

All biochemical indicators were measured using chemiluminescence methods (automated biochemical analyzer, Beckman AU5841, USA) with the same batch of reagents.

Operation of Liver Shear Wave Quantification Ultrasonography: Examinees fasted before the procedure and were positioned supine or slightly tilted to the left, with the right arm fully raised and externally rotated to widen the width of the intercostal space. Measurements were taken between the ribs, with the probe should be perpendicular to the skin surface (90°) and applying appropriate pressure. During the measurement, participants held their breath for 3–5 s in a calm state, avoiding deep inhalation.

Grading of liver fatty infiltration: mild MASLD: 240db/m ≤ CAP < 265db/m; moderate MASLD: 265db/m ≤ CAP < 295db/m; severe MASLD: CAP ≥ 295db/m. Body mass index (BMI) = weight (kg)/height^2^ (m^2^); FT3/FT4 = FT3 (pmol/L)/FT4 (pmol/L); TFQI= (cdf) FT4–(1–(cdf) TSH); TT4RI = FT4 (pmol/L) × TSH (mIU/L); TSHI=LnTSH (mIU/L) + 0.1345 × FT4 (pmol/L).

### 2.5 Definition of metabolic diseases

Hypertension: ① History of hypertension; ② systolic blood pressure ≥ 140 mmHg; ③ diastolic blood pressure ≥ 90 mmHg. Meeting any of the above conditions was sufficient. Diabetes: ① History of diabetes; ② FBG ≥ 7.0 mmol/l; ③ HbA1c ≥ 6.5%. Meeting any of the above conditions was sufficient. Impaired glucose tolerance: HbA1c ≥ 6.1 %. Hypertriglyceridemia: ① Diagnosis of or current lipid-lowering therapy for hypertriglyceridemia; ② TG ≥ 1.7 mmol/l. Meeting any of the above conditions was sufficient. Low HDL-C: HDL-C < 1.0 mmol/l.

### 2.6 Statistical analysis

We used GraphPad Prism 10.0.3, Empower(R) (; X&Y Solutions, Inc., Boston, MA), R (), and SPSS 27.0 of statistical software for data analysis. The Shapiro–Wilk test and Q–Q plots were used to assess data normality of data. Variables with normal distributions were expressed as mean ± standard deviation (Mean ± SD), while variables with skewed distributions were expressed as median (25th percentile, 75th percentile). One-way ANOVA was used to compare the differences in TH sensitivity among the normal, mild, moderate, and severe MASLD groups. The relationship between CAP and TH sensitivity was analyzed using smooth functions and threshold saturation analysis. Due to the large sample size, in the subgroup analyses of the threshold effect with statistically significant results with statistical significance were further subjected to trend tests of multiple regression equations to verify whether the correlation trends had statistical significance. All tests were conducted in a two-sided, and the statistical significance level was set at α = 0.05. Values of p < 0.05 were considered statistically significant difference.

Sample size calculation was conducted using G*Power (version 3.1.9.7). *Post hoc* power analysis indicated that, based on the observed effect size (Cohen’s f = 0.036) and the sample size (N = 13,144) in this study, the statistical power was 98% (α=0.05), suggesting extremely high statistical power (98%). The significance level (α) for this study design was set at 0.05, the expected power (1-β) was 0.80, and the required sample size was at least N = 1,082. Allowing for a 15% dropout rate, the planned minimum recruitment was 1,245 participants. Since this study utilized existing large-scale data, the final sample size (N = 13,144) greatly exceeded the minimum requirement, ensuring sufficient test power (15).

## 3 Results

### 3.1 General characteristics

A total of 13,144 individuals were included. Among them, 8,197 were male (62.36%) and 4,947 were female (37.64%). Ages ranged from 18 to 80 years old, with a mean of 43.95 ± 12.73 years. BMI ranged from 18.5 to 46.2 kg/m², with a mean of 24.94 ± 3.33 kg/m². The prevalence of chronic diseases was as follows: hyperlipidemia, 4,618 individuals (35.04%); diabetes, 1,493 individuals (11.33%); and hypertension, 5,097 individuals (38.68%).

### 3.2 One-way ANOVA on TH sensitivity among four groups graded with fatty infiltration degree in MASLD

**Figure 2:**
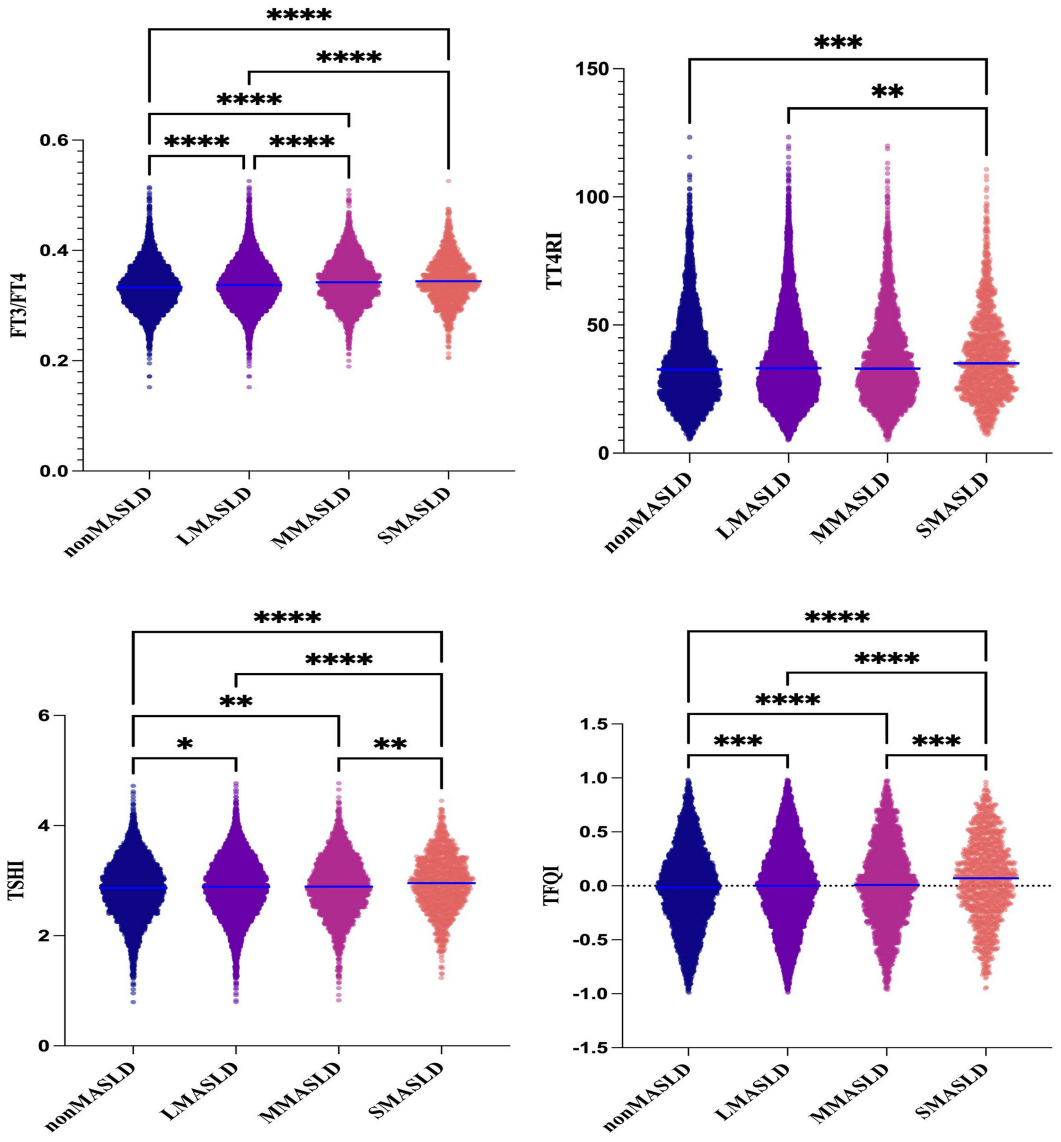
One-way ANOVA of the comparison of TH sensitivity among groups graded with fatty infiltration degree in MASLD. *P<0.05, **P<0.01, ***P<0.001, ****P<0.0001.

Subjects were divided into four groups according to CAP levels: non-MASLD, CAP <240 dB/m (N = 5,514); mild MASLD, 240 ≤ CAP <265 dB/m (N = 2,973); moderate MASLD, 265 ≤ CAP <295 dB/m (N = 3,292); and severe MASLD, CAP ≥295 dB/m (N = 1,365). With increasing severity of MASLD, FT3 and FT4 levels showed significant upward trends (p < 0.001), while TSH levels did not differ significantly across groups. In contrast, FT3/FT4, TT4RI, TSHI, and TFQI all showed significant upward trends with increasing MASLD severity (p < 0.001) (**Table 1**, **Figure 2**).

**Table 1:** One-way ANOVA of clinical data among four groups graded with fatty infiltration degree.

Group	non-MASLD	LMASLD	MMASLD	SMASLD	*P*-values
N	5514	2973	3292	1365	
Age (years)	42.561 ± 12.958	45.502 ± 12.736	45.676 ± 12.297	42.001 ± 11.880	<0.001
BMI(kg/m2)	22.655 ± 2.183	24.881 ± 2.273	26.704 ± 2.194	30.062 ± 3.128	<0.001
FBG(mmol/l)	5.170 ± 0.843	5.403 ± 1.050	5.636 ± 1.283	5.971 ± 1.737	<0.001
HbA1c (%)	5.612 ± 0.603	5.750 ± 0.682	5.884 ± 0.803	6.036 ± 0.919	<0.001
TG(mmol/l)	1.236 ± 1.342	1.685 ± 1.500	2.140 ± 2.078	2.639 ± 2.314	<0.001
LDL-C(mmol/l)	3.006 ± 0.710	3.174 ± 0.751	3.288 ± 0.730	3.308 ± 0.700	<0.001
T-CHO(mmol/l)	5.004 ± 0.974	5.163 ± 1.036	5.279 ± 1.024	5.306 ± 0.999	<0.001
HDL-C(mmol/l)	1.449 ± 0.300	1.340 ± 0.282	1.266 ± 0.259	1.182 ± 0.225	<0.001
ALT(U/L)	20.120 ± 12.858	25.052 ± 15.610	33.319 ± 21.884	51.850 ± 33.148	<0.001
AST(U/L)	22.168 ± 7.786	23.822 ± 8.500	26.620 ± 11.075	33.458 ± 16.091	<0.001
GGT(U/L)	24.068 ± 24.825	33.486 ± 36.245	43.982 ± 42.547	59.547 ± 50.683	<0.001
TIBL(umol/l)	15.013 ± 5.830	15.419 ± 5.743	15.824 ± 6.016	15.929 ± 5.878	<0.001
ALB(g/l)	45.105 ± 2.516	45.253 ± 2.488	45.550 ± 2.473	45.944 ± 2.470	<0.001
SCr(umol/l)	65.746 ± 14.539	70.504 ± 15.023	72.698 ± 14.772	73.768 ± 13.503	<0.001
SUA(umol/l)	324.167 ± 81.400	361.208 ± 86.289	391.513 ± 85.894	430.750 ± 92.769	<0.001
FT3(pmol/l)	5.287 ± 0.523	5.428 ± 0.502	5.504 ± 0.496	5.608 ± 0.472	<0.001
FT4(pmol/l)	15.958 ± 2.030	16.136 ± 2.046	16.191 ± 2.078	16.392 ± 2.049	<0.001
TSH(mIU/L)	2.277 ± 1.092	2.277 ± 1.107	2.288 ± 1.079	2.340 ± 1.062	0.277
FT3/FT4	0.335 ± 0.041	0.340 ± 0.041	0.344 ± 0.042	0.346 ± 0.042	<0.001
TT4RI	35.980 ± 17.169	36.374 ± 17.472	36.744 ± 17.441	38.101 ± 17.472	<0.001
TSHI	2.855 ± 0.522	2.877 ± 0.522	2.895 ± 0.522	2.951 ± 0.512	<0.001
TFQI	-0.024 ± 0.377	0.001 ± 0.378	0.015 ± 0.381	0.062 ± 0.376	<0.001
Gender					<0.001
Female	2990 (54.226%)	984 (33.098%)	767 (23.299%)	206 (15.092%)	
Male	2524 (45.774%)	1989 (66.902%)	2525 (76.701%)	1159 (84.908%)	
Hypertension					<0.001
No	3972 (72.035%)	1774 (59.670%)	1744 (52.977%)	582 (42.637%)	
Yes	1542 (27.965%)	1199 (40.330%)	1548 (47.023%)	783 (57.363%)	
Diabetes					<0.001
No	5095 (92.401%)	2669 (89.775%)	2815 (85.510%)	1084 (79.414%)	
Yes	419 (7.599%)	304 (10.225%)	477 (14.490%)	281 (20.586%)	
Hyperlipidemia					<0.001
No	4377 (79.380%)	1952 (65.658%)	1680 (51.033%)	527 (38.608%)	
Yes	1137 (20.620%)	1021 (34.342%)	1612 (48.967%)	838 (61.392%)	

Result: Mean ± Standard Deviation/N (%); P-value: Kruskal Wallis rank sum test is used to obtain the result for continuous variables.

### 3.3 Smooth fitting curve and threshold saturation analysis of the relationship between CAP and TH sensitivity

**Figure 3:**
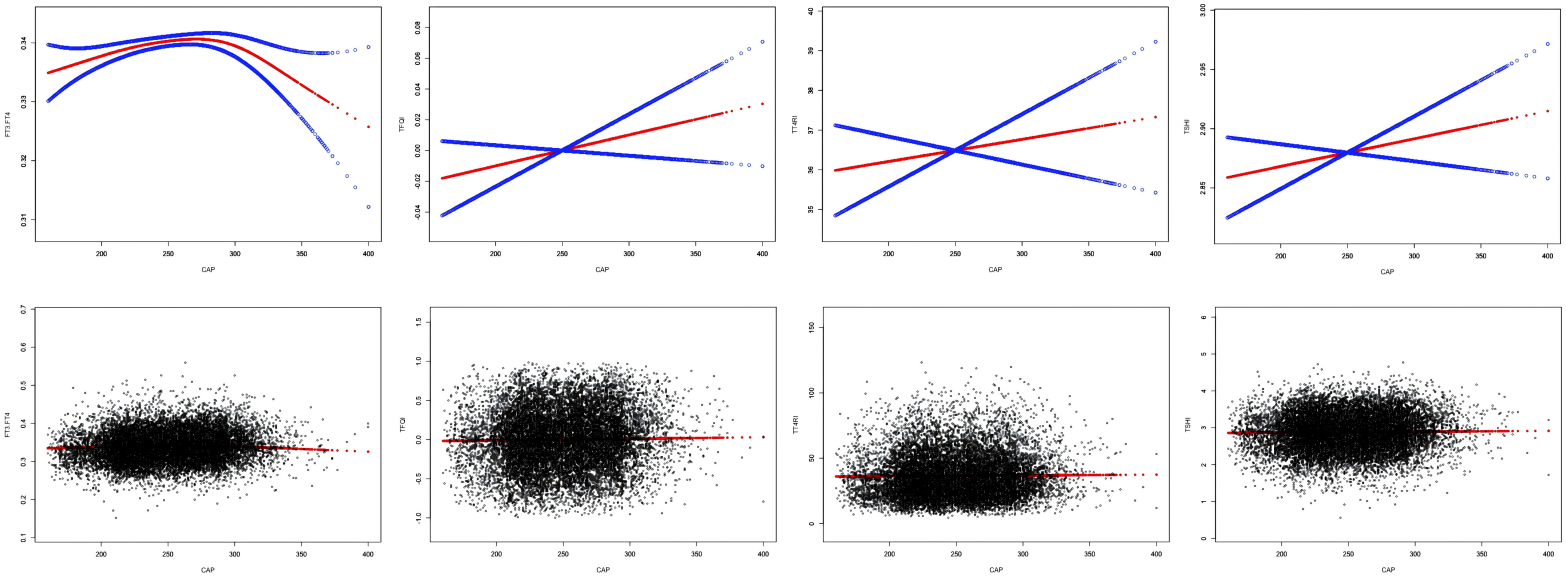
Smooth fitting curve of the relationship between CAP and TH sensitivity.

After adjusting for sex, age, BMI, hypertension, diabetes, hyperlipidemia, FBG, HbA1c, TG, T-CHO, LDL-C, HDL-C, ALT, AST, GGT, TBIL, ALB, SCr, and SUA, CAP was positively correlated with FT3/FT4 before the inflection point at CAP = 294 dB/m (β = 0.000, p = 0.0029) but was negatively correlated with FT3/FT4 after the inflection point (β = −0.000, p = 0.0002). No significant correlations were observed between CAP and TT4RI, TSHI, or TFQI (p > 0.05) (**Table 2**, **Figure 3**).

**Table 2:** Threshold effect analysis of the relationship between CAP and TH sensitivity.

For exposure: CAP				
Outcome:	FT3/FT4	TT4RI	TSHI	TFQI
Model I				
One linear effect	0.000 (-0.000, 0.000) 0.0761	0.005 (-0.008, 0.018) 0.4594	0.000 (-0.000, 0.001) 0.3357	0.000 (-0.000, 0.000) 0.2672
Model II				
inflection point(K)	294	272	272	272
< K Segment Effect 1	0.000 (0.000, 0.000) 0.0029	0.001 (-0.014, 0.015) 0.9441	0.000 (-0.000, 0.000) 0.8422	0.000 (-0.000, 0.000) 0.7556
> K Segment Effect 2	-0.000 (-0.000, -0.000) 0.0002	0.021 (-0.009, 0.051) 0.1733	0.001 (-0.000, 0.002) 0.1153	0.001 (-0.000, 0.001) 0.0958
Effect difference	-0.000 (-0.000, -0.000) <0.0001	0.020 (-0.014, 0.054) 0.2475	0.001 (-0.000, 0.002) 0.1981	0.000 (-0.000, 0.001) 0.1874
The predicted value of the equation at the inflection point	0.346 (0.345, 0.347)	36.505 (35.974, 37.037)	2.886 (2.870, 2.902)	0.009 (-0.002, 0.021)
Log-likelihood ratio test	<0.001	0.247	0.198	0.187

Outcome: β (95%CI) *P*-value; β: regression coefficient, CI confidence interval; result variable: FT3/FT4, TT4RI, TSHI, TFQI; exposed variable: CAP; adjusted for gender, age, BMI, hypertension, diabetes, hyperlipidemia, FBG, HbA1c, TG, T-CHO, LDL-C, HDL-C, ALT, AST, GGT, TBIL, ALB, SCr, SUA.

### 3.4 The trend tests of multiple regression equations for the relationship between CAP and TH sensitivity

The smooth fitting curve and threshold saturation analysis revealed that CAP was positively correlated with FT3/FT4 before the inflection point (CAP = 294 dB/m) but negatively correlated after the inflection point. To verify whether these correlation trends had statistical significance, trend tests of multiple regression equations were performed. Trend p values were statistically analyzed using CAP quartiles as continuous variables. Model I adjusted for gender, age, and BMI. Model II adjusted for gender, age, BMI, hypertension, diabetes, hyperlipidemia, FBG, HbA1c, TG, T-CHO, LDL-C, HDL-C, ALT, AST, GGT, TBIL, ALB, SCr, and SUA.

**Figure 4:**
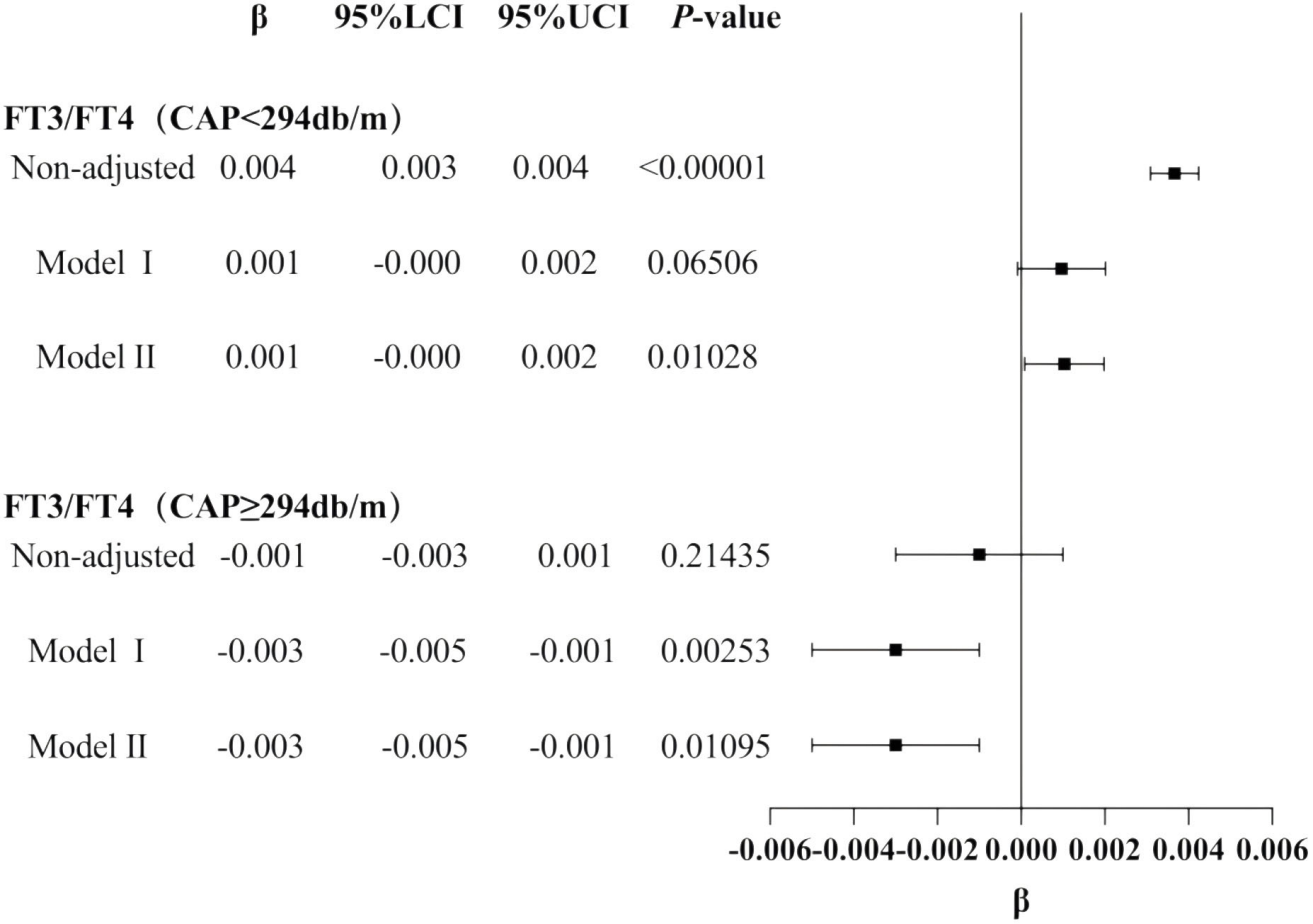
Forest map for the trend test results of CAP and TH sensitivity.

The results of the trend test for multiple regression equations between CAP and FT3/FT4 in individuals with CAP<294db/m (N = 11665) showed that as the CAP quartiles levels increased, the trend of gradually increasing FT3/FT4 had no statistical significance in Model I (*P* = 0.06506). In individuals with CAP <294 dB/m (N = 11,665), as CAP quartile levels increased, the trend of gradually increasing FT3/FT4 did not reach statistical significance in Model I (p = 0.06506). In individuals with CAP ≥294 dB/m (N = 1,479), before adjusting for confounding factors, there was no statistically significant trend of gradually decreasing FT3/FT4 with increasing CAP quartile levels (p = 0.21435) (**Table 3**, **Figure 4**). Considering that CAP and THs are significantly influenced by sex, age, and BMI, subgroup analyses were further conducted based on these variables.

**Table 3:** Trend analysis of multiple regression equations of CAP and FT3/FT4.

Exposure	Non-adjusted	*P*-value	Model I	*P*-value	Model II	*P*-value
	β (95%CI)		β (95%CI)		β (95%CI)	
FT3/FT4(CAP<294db/m)						
CAP	0.000 (0.000, 0.000)	<0.0001	0.000 (0.000, 0.000)	0.0406	0.000 (0.000, 0.000)	0.0065
CAP quartile						
Q1	0		0		0	
Q2	0.005 (0.003, 0.007)	0.00001	0.002 (-0.000, 0.004)	0.08052	0.002 (0.000, 0.004)	0.04966
Q3	0.008 (0.005, 0.010)	<0.00001	0.002 (-0.000, 0.005)	0.06333	0.003 (0.001, 0.005)	0.01381
Q4	0.011 (0.009, 0.013)	<0.00001	0.003 (-0.000, 0.005)	0.05625	0.004 (0.001, 0.006)	0.01091
CAP quartile continuous	0.004 (0.003, 0.004)		0.001 (-0.000, 0.002)		0.001 (0.000, 0.002)	
*P* for trend	<0.00001		0.06506		0.01028	
FT3/FT4(CAP≥294db/m)						
CAP	-0.000 (-0.000, 0.000)	0.14079	-0.000 (-0.000, -0.000)	0.00089	-0.000 (-0.000, -0.000)	0.00197
CAP quartile						
Q1	0		0		0	
Q2	0.001 (-0.005, 0.007)	0.78534	0.000 (-0.006, 0.006)	0.99527	0.000 (-0.006, 0.006)	0.94121
Q3	0.001 (-0.005, 0.007)	0.66009	-0.001 (-0.007, 0.005)	0.75078	0.000 (-0.006, 0.006)	0.92552
Q4	-0.004 (-0.010, 0.002)	0.18003	-0.011 (-0.017, -0.004)	0.00134	-0.009 (-0.015, -0.003)	0.00485
CAP quartile continuous	-0.001 (-0.003, 0.001)		-0.003 (-0.005, -0.001)		-0.003 (-0.005, -0.001)	
*P* for trend	0.21435		0.00253		0.01095	

Outcome: β (95%CI) *P*-value, β: regression coefficient, CI Confidence interval; Result variable: FT3/FT4, Exposed variable: CAP; Model I: Adjusted for gender, age, BMIModel II: Adjusted for gender, age, BMI, hypertension, diabetes, hyperlipidemia, FBG, HbA1c, TG, T-CHO, LDL-C, HDL-C, ALT, AST, GGT, TBIL, ALB, SCr, SUA.

### 3.5 Smooth fitting curve and threshold saturation analysis of the relationship between CAP and TH sensitivity in gender subgroups

In females, after adjusting for age, BMI, hypertension, diabetes, hyperlipidemia, FBG, HbA1c, TG, T-CHO, LDL-C, HDL-C, ALT, AST, GGT, TBIL, ALB, SCr, and SUA, CAP showed no correlation with FT3/FT4, TSHI, TFQI, or TT4RI (p > 0.05).

**Figure 5:**
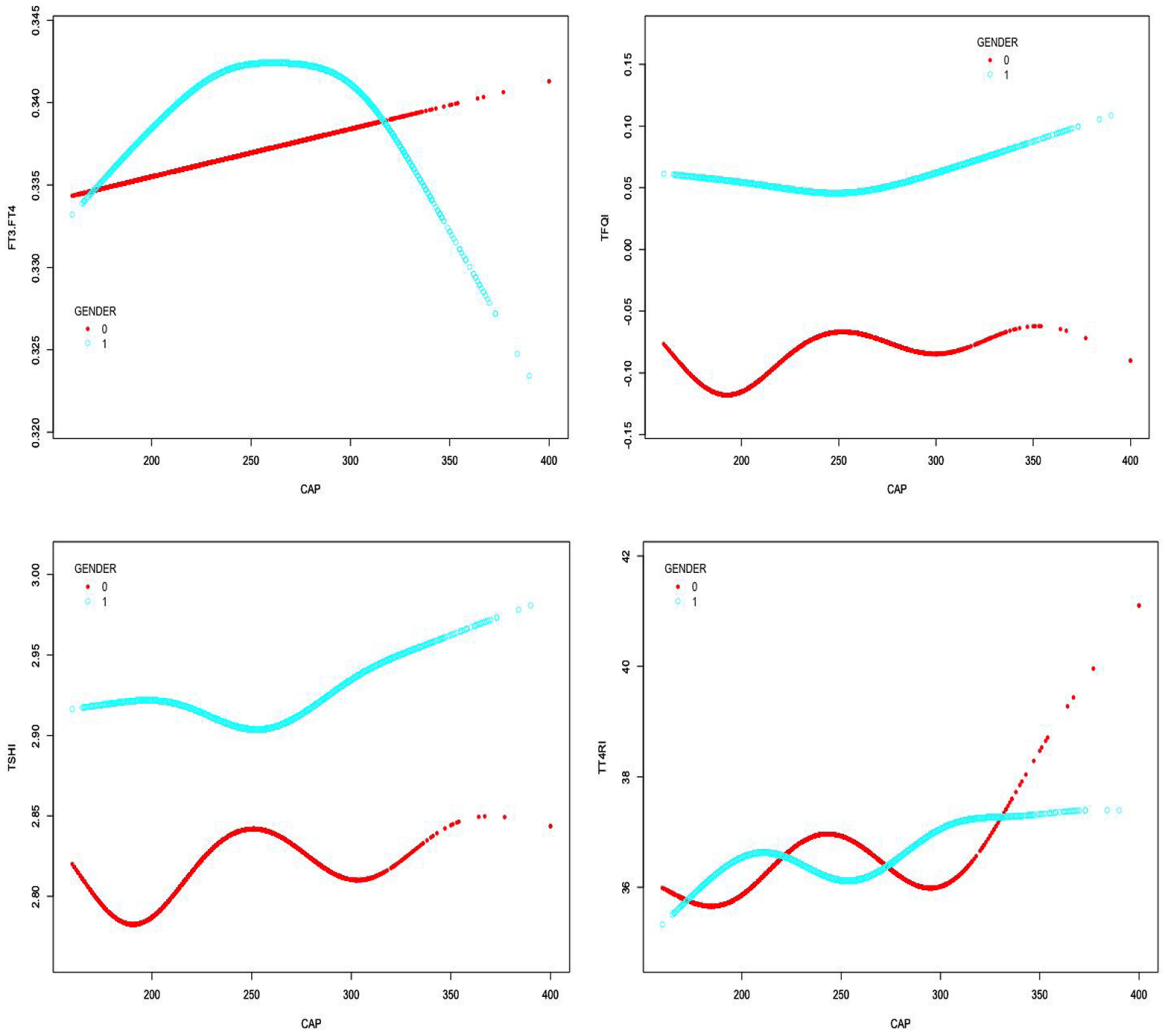
Smooth-fitting curves of the relationship between CAP and TH sensitivity in gender subgroup (0 for females, 1 for males).

In males, CAP was not correlated with FT3/FT4 before the inflection point at CAP = 305 dB/m but was negatively correlated with FT3/FT4 after the inflection point (β = −0.000, p < 0.0001). CAP was not correlated with TSHI before the inflection point at CAP = 252 dB/m but was positively correlated with TSHI after the inflection point: for each unit increase in CAP, TSHI increased by 0.001 (β = 0.001, p = 0.0067). Similarly, CAP was not correlated with TFQI before the inflection point at CAP = 252 dB/m but was positively correlated with TFQI after the inflection point: for each unit increase in CAP, TFQI increased by 0.001 (β = 0.001, p = 0.0022). No correlation was observed between CAP and TT4RI (p > 0.05) (**Table 4**, **Figure 5**).

**Table 4:** Threshold effect analysis of the relationship between CAP and TH sensitivity in gender subgroup.

For exposure: CAP	FT3/FT4		TT4RI	
Outcome:	Female	Male	Female	Male
Model I				
One linear effect	0.000 (0.000, 0.000) 0.0426	0.000 (-0.000, 0.000) 0.8179	-0.004 (-0.024, 0.017) 0.7096	0.010 (-0.006, 0.026) 0.2186
Model II				
inflection point(K)	276	305	226	268
< K Segment Effect 1	0.000 (0.000, 0.000) 0.0143	0.000 (-0.000, 0.000) 0.1810	0.019 (-0.018, 0.057) 0.3124	0.001 (-0.020, 0.021) 0.9313
> K Segment Effect 2	-0.000 (-0.000, 0.000) 0.3658	-0.000 (-0.001, -0.000) <0.0001	-0.019 (-0.049, 0.010) 0.1946	0.031 (-0.001, 0.064) 0.0613
Effect difference	-0.000 (-0.000, 0.000) 0.1177	-0.000 (-0.001, -0.000) <0.0001	-0.039 (-0.092, 0.014) 0.1482	0.030 (-0.011, 0.071) 0.1465
The predicted value of the equation at the inflection point	0.343 (0.340, 0.345)	0.347 (0.346, 0.349)	36.547 (35.762, 37.333)	36.181 (35.528, 36.834)
Log-likelihood ratio test	0.117	<0.001	0.148	0.146
For exposure: CAP	TSHI		TFQI	
Outcome:	Female	Male	Female	Male
Model I				
One linear effect	-0.000 (-0.001, 0.000) 0.6976	0.000 (-0.000, 0.001) 0.1192	-0.000 (-0.001, 0.000) 0.5580	0.000 (-0.000, 0.001) 0.0662
Model II				
inflection point(K)	228	252	183	252
< K Segment Effect 1	0.001 (-0.000, 0.002) 0.2883	-0.000 (-0.001, 0.000) 0.4392	-0.004 (-0.008, 0.001) 0.1281	-0.000 (-0.001, 0.000) 0.4367
> K Segment Effect 2	-0.001 (-0.002, 0.000) 0.1606	0.001 (0.000, 0.002) 0.0067	-0.000 (-0.000, 0.000) 0.9805	0.001 (0.000, 0.001) 0.0022
Effect difference	-0.001 (-0.003, 0.000) 0.1203	0.001 (0.000, 0.003) 0.0259	0.004 (-0.001, 0.008) 0.1408	0.001 (0.000, 0.002) 0.0141
The predicted value of the equation at the inflection point	2.825 (2.802, 2.849)	2.888 (2.868, 2.908)	-0.093 (-0.113, -0.073)	0.031 (0.017, 0.046)
Log-likelihood ratio test	0.12	0.026	0.14	0.014

Outcome: β (95%CI) *P*-value; β: regression coefficient, CI Confidence interval; Result variable: FT3/FT4, TT4RI, TSHI, TFQI; Exposed variable: CAP; Adjusted for age, BMI, hypertension, diabetes, hyperlipidemia, FBG, HbA1c, TG, T-CHO, LDL-C, HDL-C, ALT, AST, GGT, TBIL, ALB, SCr, SUA.

### 3.6 Trend tests of multiple regression equations for the relationship between CAP and thyroid hormone sensitivity in gender subgroups

The results for the male group with CAP ≥305 dB/m (N = 687) of trend tests of multiple regression equations between CAP and FT3/FT4 showed that as the CAP quartiles increased, FT3/FT4 decreased significantly; for each increased quartile in CAP, FT3/FT4 decreased by 0.005 (β = −0.005, p = 0.0004).

The results for the male group with CAP ≥252 dB/m (N = 4,823) showed that as the CAP quartiles increased, TSHI showed that as the CAP quartiles increased, the trend of gradually increased significantly; for each increased quartile in CAP, TSHI increased by 0.019 (β = 0.019, p = 0.0248).

**Figure 6:**
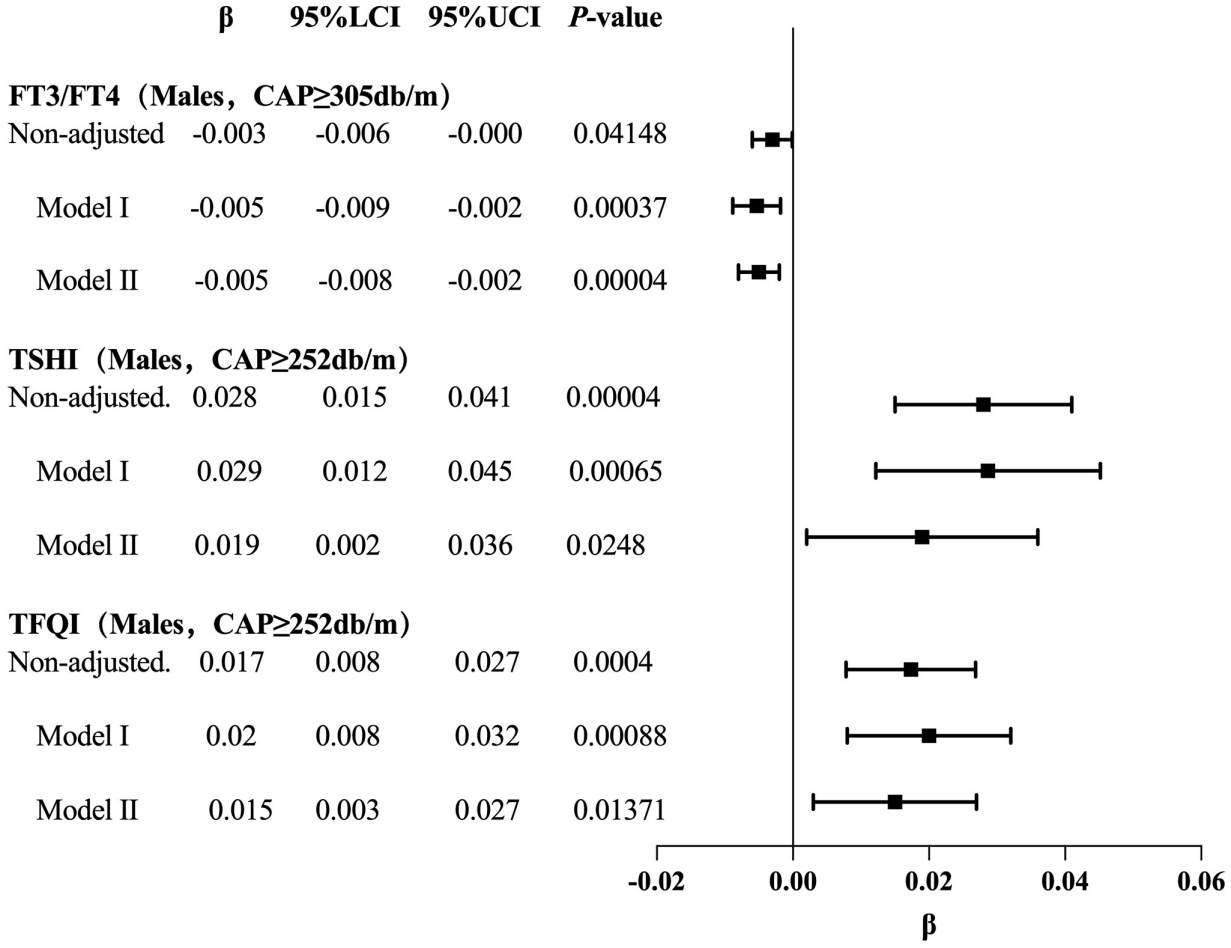
Forest map for the trend test results of CAP and TH sensitivity in gender subgroup.

The results for the male group with CAP ≥252 dB/m (N = 4,823) also showed that as the CAP quartiles increased, TFQI increased significantly; for each increased quartile in CAP, TFQI increased by 0.015 (β = 0.015, p = 0.01371) (**Table 5**, **Figure 6**).

**Table 5:** Trend analysis of multiple regression equations for gender subgroups of CAP and thyroid hormone sensitivity.

Exposure	Non-adjusted	*P*-value	Model I	*P*-value	Model II	*P*-value
	β (95%CI)		β (95%CI)		β (95%CI)	
FT3/FT4 (Males, CAP≥305db/m)						
CAP	-0.000 (-0.000, 0.000)	0.05554	-0.000 (-0.001, -0.000)	0.0012	-0.000 (-0.001, -0.000)	0.00046
CAP quartile						
Q1	0		0		0	
Q2	-0.001 (-0.010, 0.008)	0.82935	-0.002 (-0.011, 0.007)	0.68894	-0.002 (-0.011, 0.007)	0.69127
Q3	-0.004 (-0.013, 0.006)	0.41951	-0.008 (-0.017, 0.002)	0.10755	-0.007 (-0.017, 0.002)	0.11572
Q4	-0.009 (-0.018, 0.000)	0.06359	-0.016 (-0.025, -0.006)	0.00117	-0.015 (-0.025, -0.006)	0.00123
CAP quartile continuous	-0.003 (-0.006, -0.000)		-0.005 (-0.009, -0.002)		-0.005 (-0.008, -0.002)	
*P* for trend	0.04148		0.00037		0.0004	
TSHI (Males, CAP≥252db/m)						
CAP	0.001 (0.001, 0.002)	0.00004	0.002 (0.001, 0.002)	0.00087	0.001 (0.000, 0.002)	0.03328
CAP quartile						
Q1	0		0		0	
Q2	-0.002 (-0.045, 0.040)	0.92458	0.001 (-0.041, 0.044)	0.94972	-0.007 (-0.049, 0.036)	0.75506
Q3	0.051 (0.009, 0.093)	0.01684	0.059 (0.015, 0.103)	0.00919	0.042 (-0.002, 0.086)	0.06242
Q4	0.075 (0.033, 0.117)	0.00052	0.076 (0.025, 0.128)	0.00386	0.048 (-0.005, 0.101)	0.07693
CAP quartile continuous	0.028 (0.015, 0.041)		0.029 (0.012, 0.045)		0.019 (0.002, 0.036)	
*P* for trend	0.00004		0.00065		0.0248	
TFQI (Males, CAP≥252db/m)						
CAP	0.001 (0.000, 0.001)	0.00019	0.001 (0.001, 0.002)	0.00037	0.001 (0.000, 0.002)	0.00742
CAP quartile						
Q1	0		0		0	
Q2	-0.005 (-0.036, 0.026)	0.75877	0.000 (-0.030, 0.031)	0.99039	-0.004 (-0.035, 0.026)	0.77919
Q3	0.032 (0.001, 0.062)	0.04195	0.043 (0.011, 0.075)	0.00817	0.034 (0.002, 0.066)	0.03615
Q4	0.045 (0.015, 0.076)	0.00364	0.052 (0.015, 0.090)	0.00575	0.038 (-0.000, 0.076)	0.05283
CAP quartile continuous	0.017 (0.008, 0.027)		0.020 (0.008, 0.032)		0.015 (0.003, 0.027)	
*P* for trend	0.0004		0.00088		0.01371	

Outcome: β (95%CI) *P*-value, β: regression coefficient, CI Confidence interval; Result variable: FT3/FT4, TSHI, TFQI, Exposed variable: CAP; Model I: Adjusted for age, BMI; Model II: Adjusted for age, BMI, hypertension, diabetes, hyperlipidemia, FBG, HbA1c, TG, T-CHO, LDL-C, HDL-C, ALT, AST, GGT, TBIL, ALB, SCr, SUA.

### 3.7 Smooth fitting curve and threshold saturation analysis of the relationship between CAP and TH sensitivity in age subgroup

In the age <65 years group, after adjusting for gender, BMI, hypertension, diabetes, hyperlipidemia, FBG, HbA1c, TG, T-CHO, LDL-C, HDL-C, ALT, AST, GGT, TBIL, ALB, SCr, and SUA, CAP was positively correlated with FT3/FT4 before the inflection point at CAP = 277 dB/m (β = 0.000, p = 0.0011) but was negatively correlated with FT3/FT4 after the inflection point (β = −0.000, p = 0.0003). CAP was not correlated with TSHI before the inflection point at CAP = 272 dB/m, but was positively correlated with TSHI after the inflection point: for each unit increase in CAP, TSHI increased by 0.001 (β = 0.001, p = 0.0287). CAP was not correlated with TFQI before the inflection point at CAP = 270 dB/m, but was positively correlated with TFQI after the inflection point: for each unit increase in CAP, TFQI increased by 0.001 (β = 0.001, p = 0.0035). There was no correlation between CAP and TT4RI.

**Figure 7:**
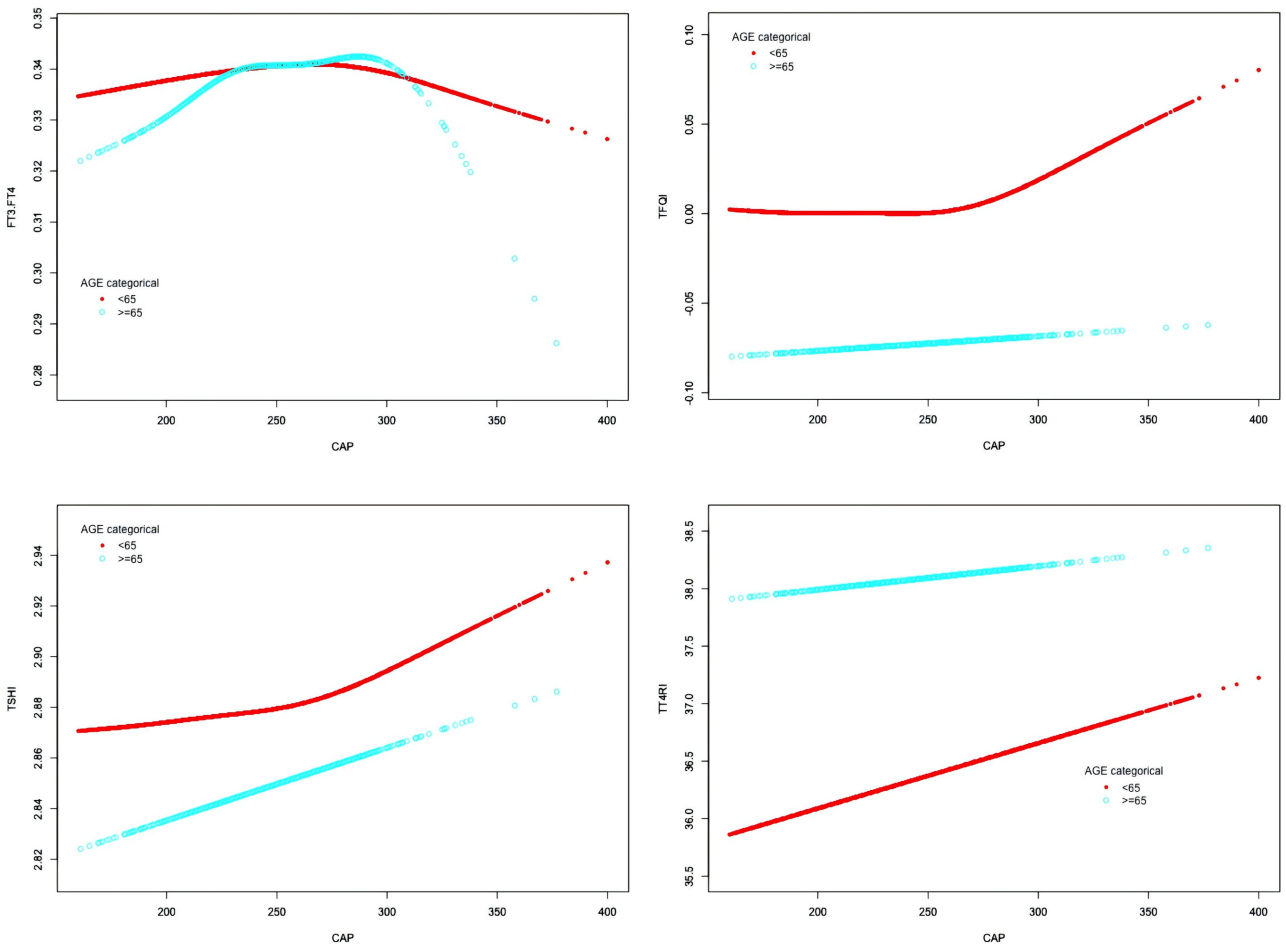
Smooth-fitting curves of the relationship between CAP and thyroid hormone sensitivity in age subgroup.

In the age ≥65 years group, CAP was not correlated with FT3/FT4 before the inflection point at CAP = 295 dB/m, but was negatively correlated with FT3/FT4 after the inflection point: for each unit increase in CAP, FT3/FT4 decreased by 0.001 (β = −0.001, p = 0.0024). CAP was not correlated with TFQI before the inflection point at CAP = 294 dB/m, but was positively correlated with TFQI after the inflection point: for each unit increase in CAP, TFQI increased by 0.004 (β = 0.004, p = 0.047). There were no correlations between CAP and TSHI or TT4RI (**Table 6**, **Figure 7**).

**Table 6:** Threshold effect analysis of the relationship between CAP and TH sensitivity in age subgroup.

For exposure: CAP	FT3/FT4		TT4RI	
Outcome:	<65	≥65	<65	≥65
Model I				
One linear effect	0.000 (-0.000, 0.000) 0.1575	0.000 (-0.000, 0.000) 0.4538	0.006 (-0.007, 0.019) 0.3967	-0.001 (-0.051, 0.050) 0.9824
Model II				
inflection point(K)	277	295	272	196
< K Segment Effect 1	0.000 (0.000, 0.000) 0.0011	0.000 (-0.000, 0.000) 0.0752	0.002 (-0.013, 0.017) 0.8346	0.394 (-0.001, 0.788) 0.0508
> K Segment Effect 2	-0.000 (-0.000, -0.000) 0.0003	-0.001 (-0.001, -0.000) 0.0024	0.021 (-0.010, 0.051) 0.1822	-0.023 (-0.078, 0.033) 0.4241
Effect difference	-0.000 (-0.000, -0.000) <0.0001	-0.001 (-0.002, -0.000) 0.0010	0.019 (-0.016, 0.054) 0.2831	-0.416 (-0.829, -0.003) 0.0486
The predicted value of the equation at the inflection point	0.344 (0.342, 0.345)	0.349 (0.343, 0.355)	36.402 (35.853, 36.950)	38.167 (35.484, 40.850)
Log-likelihood ratio test	<0.001	<0.001	0.283	0.047
For exposure: CAP	TSHI		TFQI	
Outcome:	<65	≥65	<65	≥65
Model I				
One linear effect	0.000 (-0.000, 0.001) 0.3743	0.000 (-0.001, 0.002) 0.8174	0.000 (-0.000, 0.000) 0.3364	-0.000 (-0.001, 0.001) 0.9893
Model II				
inflection point(K)	272	196	270	294
< K Segment Effect 1	-0.000 (-0.001, 0.000) 0.8319	0.010 (-0.001, 0.021) 0.0867	-0.000 (-0.000, 0.000) 0.5426	-0.000 (-0.001, 0.001) 0.4979
> K Segment Effect 2	0.001 (0.000, 0.002) 0.0287	-0.000 (-0.002, 0.001) 0.6470	0.001 (0.000, 0.002) 0.0035	0.004 (0.000, 0.009) 0.0470
Effect difference	0.001 (0.000, 0.002) 0.0454	-0.010 (-0.022, 0.002) 0.0897	0.001 (0.000, 0.002) 0.0054	0.005 (0.000, 0.009) 0.0408
The predicted value of the equation at the inflection point	2.891 (2.874, 2.907)	2.841 (2.763, 2.918)	0.015 (0.003, 0.027)	-0.086 (-0.134, -0.038)
Log-likelihood ratio test	0.045	0.088	0.005	0.039

Outcome: β (95%CI) *P*-value; β: regression coefficient, CI Confidence interval; Result variable: FT3/FT4, TT4RI, TSHI, TFQI; Exposed variable: CAP; Adjusted for gender, BMI, hypertension, diabetes, hyperlipidemia, FBG, HbA1c, TG, T-CHO, LDL-C, HDL-C, ALT, AST, GGT, TBIL, ALB, SCr, SUA.

### 3.8 The trend tests of multiple regression equations for the relationship between CAP and TH sensitivity in age subgroup

For the age <65 years group with CAP <277 dB/m (N = 9,193), trend analysis of multiple regression equations between CAP and FT3/FT4 showed that as the CAP quartiles increased, the trend of gradually increasing FT3/FT4 was not statistically significant. For the age <65 years group with CAP ≥277 dB/m (N = 3,121), trend analysis of multiple regression equations between CAP and FT3/FT4 showed that as the CAP quartiles increased, the trend of gradually decreasing FT3/FT4 was not statistically significant.

For the age ≥65 years group with CAP ≥295 dB/m (N = 49) and the age ≥65 years group with CAP ≥294 dB/m (N = 52), the small sample size led to statistical bias; therefore, no analysis was conducted for these groups.

For the age <65 years group with CAP ≥272 dB/m (N = 3,569), trend analysis of multiple regression equations between CAP and TSHI showed that as the CAP quartiles increased, the trend of gradually increasing TSHI was not statistically significant.

**Figure 8:**
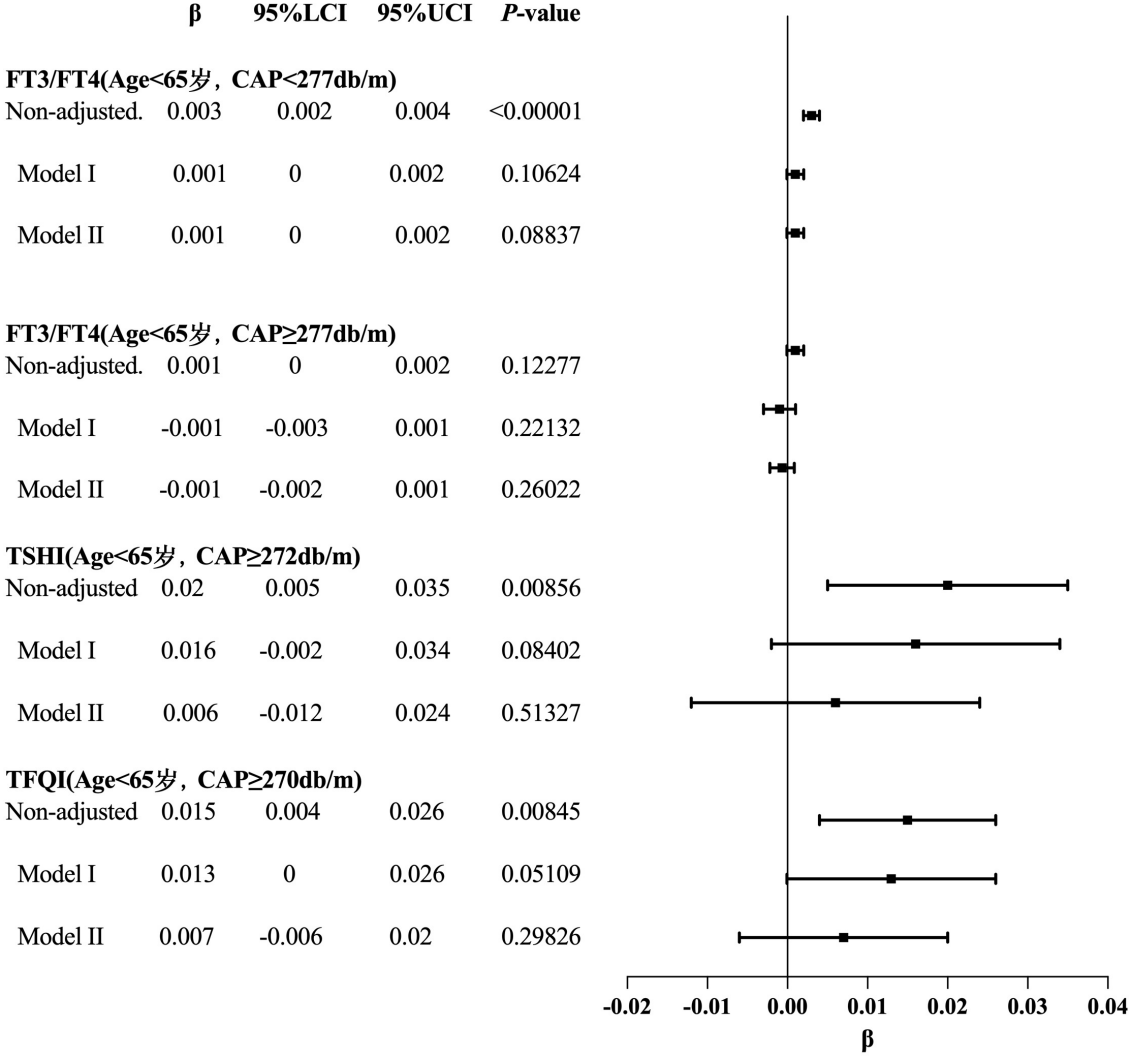
Forest map for the trend test results of CAP and TH sensitivity in age subgroup.

For the age <65 years group with CAP ≥270 dB/m (N = 3,871), trend analysis of multiple regression equations between CAP and TFQI showed that as the CAP quartiles increased, the trend of gradually increasing TFQI was not statistically significant (**Table 7**, **Figure 8**).

**Table 7:** Trend analysis of multiple regression equations of the relationship between CAP and thyroid hormone sensitivity indicators in age subgroups.

Exposure	Non-adjusted	*P*-value	Model I	*P*-value	Model II	*P*-value
	β (95%CI)		β (95%CI)		β (95%CI)	
FT3/FT4(Age<65 years, CAP<277db/m)						
CAP	0.000 (0.000, 0.000)	<0.00001	0.000 (0.000, 0.000)	0.03004	0.000 (0.000, 0.000)	0.02714
CAP quartile						
Q1	0		0		0	
Q2	0.003 (0.000, 0.005)	0.01981	0.001 (-0.002, 0.003)	0.59533	0.001 (-0.002, 0.003)	0.56235
Q3	0.007 (0.005, 0.010)	<0.00001	0.002 (-0.000, 0.005)	0.06697	0.003 (0.000, 0.005)	0.04875
Q4	0.008 (0.006, 0.011)	<0.00001	0.002 (-0.001, 0.005)	0.16961	0.002 (-0.001, 0.005)	0.14847
CAP quartile continuous	0.003 (0.002, 0.004)		0.001 (-0.000, 0.002)		0.001 (-0.000, 0.002)	
*P* for trend	<0.00001		0.10624		0.08837	
FT3/FT4(Age<65 years, CAP≥277db/m)						
CAP	0.000 (-0.000, 0.000)	0.29595	-0.000 (-0.000, -0.000)	0.03111	-0.000 (-0.000, -0.000)	0.02848
CAP quartile						
Q1	0		0		0	
Q2	0.000 (-0.004, 0.005)	0.86614	-0.001 (-0.005, 0.003)	0.61293	-0.001 (-0.005, 0.003)	0.63681
Q3	0.002 (-0.002, 0.006)	0.3017	-0.001 (-0.005, 0.004)	0.7427	-0.001 (-0.005, 0.004)	0.73001
Q4	0.003 (-0.001, 0.007)	0.18462	-0.003 (-0.008, 0.001)	0.1642	-0.003 (-0.008, 0.002)	0.20461
CAP quartile continuous	0.001 (-0.000, 0.002)		-0.001 (-0.003, 0.001)		-0.001 (-0.002, 0.001)	
*P* for trend	0.12277		0.22132		0.26022	
TSHI(Age<65 years, CAP≥272db/m)						
CAP	0.001 (0.000, 0.002)	0.01308	0.001 (-0.000, 0.002)	0.11407	0.000 (-0.001, 0.002)	0.52522
CAP quartile						
Q1	0		0		0	
Q2	0.067 (0.019, 0.116)	0.00676	0.065 (0.016, 0.113)	0.00915	0.061 (0.012, 0.109)	0.01368
Q3	0.041 (-0.006, 0.089)	0.08599	0.034 (-0.015, 0.083)	0.17214	0.018 (-0.032, 0.067)	0.48488
Q4	0.076 (0.029, 0.124)	0.00169	0.062 (0.006, 0.118)	0.03052	0.033 (-0.024, 0.090)	0.26203
CAP quartile continuous	0.020 (0.005, 0.035)		0.016 (-0.002, 0.034)		0.006 (-0.012, 0.024)	
*P* for trend	0.00856		0.08402		0.51327	
TFQI(Age<65 years, CAP≥270db/m)						
CAP	0.001 (0.000, 0.002)	0.00495	0.001 (0.000, 0.002)	0.0228	0.001 (-0.000, 0.001)	0.14681
CAP quartile						
Q1	0		0		0	
Q2	0.052 (0.018, 0.087)	0.00303	0.050 (0.016, 0.084)	0.00427	0.049 (0.015, 0.083)	0.0051
Q3	0.035 (0.000, 0.069)	0.04773	0.031 (-0.004, 0.066)	0.08422	0.021 (-0.014, 0.057)	0.23277
Q4	0.057 (0.022, 0.091)	0.00135	0.049 (0.009, 0.089)	0.01752	0.031 (-0.010, 0.072)	0.1368
CAP quartile continuous	0.015 (0.004, 0.026)		0.013 (-0.000, 0.026)		0.007 (-0.006, 0.020)	
*P* for trend	0.00845		0.05109		0.29826	

Outcome: β (95%CI) *P*-value, β: regression coefficient, CI Confidence interval; Result variable: FT3/FT4, TSHI, TFQI, Exposed variable: CAP; Model I: Adjusted for gender, BMI; Model II: Adjusted for gender, BMI, hypertension, diabetes, hyperlipidemia, FBG, HbA1c, TG, T-CHO, LDL-C, HDL-C, ALT, AST, GGT, TBIL, ALB, SCr, SUA.

### 3.9 Smooth fitting curve and threshold saturation analysis of the relationship between CAP and TH sensitivity in BMI subgroups

**Figure 9:**
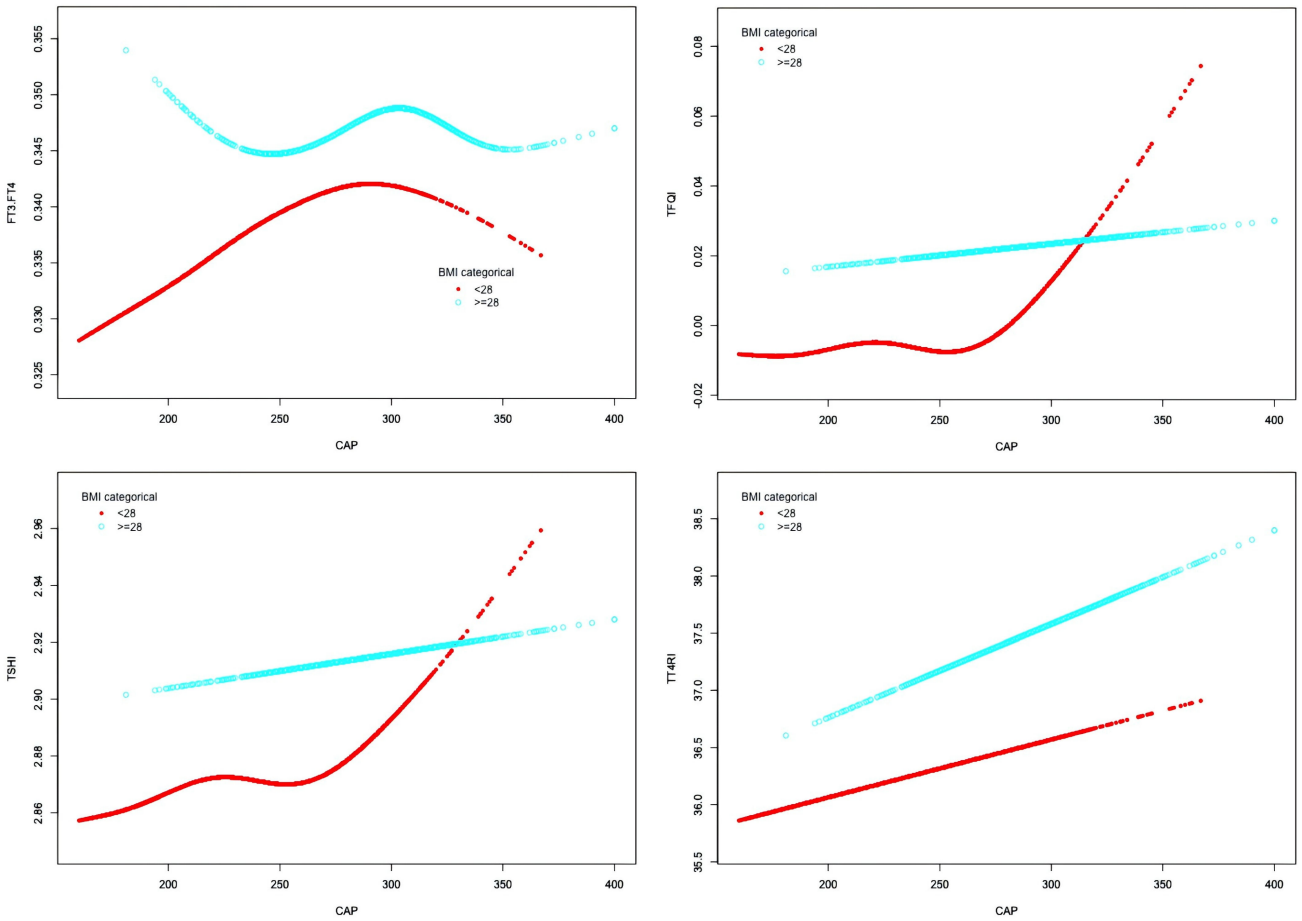
Smooth-fitting curves of the association between CAP and thyroid hormone sensitivity in BMI subgroup.

In the BMI <28 kg/m² group, after adjusting for gender, age, hypertension, diabetes, hyperlipidemia, FBG, HbA1c, TG, T-CHO, LDL-C, HDL-C, ALT, AST, GGT, TBIL, ALB, SCr, and SUA, CAP was positively correlated with FT3/FT4 before the inflection point at CAP = 277 dB/m (β = 0.000, p < 0.0001) but was not correlated with FT3/FT4 after the inflection point. CAP was not correlated with TFQI before the inflection point at CAP = 275 dB/m, but was positively correlated with TFQI after the inflection point: for each unit increase in CAP, TFQI increased by 0.001 (β = 0.001, p = 0.0136). There was no correlation between CAP and TSHI or TT4RI (**Table 8**, **Figure 9**).

**Table 8:** Threshold effect analysis of BMI subgroup analysis of the relationship between CAP and thyroid hormone sensitivity indicators.

For exposure: CAP	FT3/FT4		TT4RI	
Outcome:	<28	≥28	<28	≥28
Model I				
One linear effect	0.000 (0.000, 0.000) <0.0001	0.000 (-0.000, 0.000) 0.2413	0.005 (-0.007, 0.016) 0.4370	0.004 (-0.025, 0.034) 0.7695
Model II				
inflection point(K)	277	309	275	255
< K Segment Effect 1	0.000 (0.000, 0.000) <0.0001	0.000 (-0.000, 0.000) 0.0553	-0.000 (-0.014, 0.013) 0.9861	0.064 (-0.052, 0.180) 0.2767
> K Segment Effect 2	-0.000 (-0.000, 0.000) 0.0707	-0.000 (-0.000, 0.000) 0.2637	0.040 (-0.012, 0.092) 0.1326	-0.007 (-0.043, 0.029) 0.7055
Effect difference	-0.000 (-0.000, -0.000) 0.0009	-0.000 (-0.000, 0.000) 0.1065	0.040 (-0.018, 0.098) 0.1723	-0.071 (-0.205, 0.062) 0.2947
The predicted value of the equation at the inflection point	0.343 (0.342, 0.345)	0.349 (0.347, 0.352)	36.426 (35.829, 37.024)	37.048 (35.494, 38.602)
Log-likelihood ratio test	<0.001	0.106	0.172	0.294
For exposure: CAP	TSHI		TFQI	
Outcome:	<28	≥28	<28	≥28
Model I				
One linear effect	0.000 (-0.000, 0.001) 0.3903	0.000 (-0.001, 0.001) 0.9984	0.000 (-0.000, 0.000) 0.5033	-0.000 (-0.001, 0.001) 0.9767
Model II				
inflection point(K)	272	249	275	249
< K Segment Effect 1	-0.000 (-0.000, 0.000) 0.7583	0.002 (-0.002, 0.006) 0.2853	-0.000 (-0.000, 0.000) 0.5338	0.002 (-0.001, 0.005) 0.2832
> K Segment Effect 2	0.001 (0.000, 0.003) 0.0413	-0.000 (-0.001, 0.001) 0.5666	0.001 (0.000, 0.003) 0.0136	-0.000 (-0.001, 0.001) 0.5447
Effect difference	0.002 (-0.000, 0.003) 0.0590	-0.002 (-0.007, 0.002) 0.2744	0.001 (0.000, 0.003) 0.0174	-0.002 (-0.005, 0.001) 0.2694
The predicted value of the equation at the inflection point	2.882 (2.864, 2.900)	2.883 (2.834, 2.931)	0.011 (-0.002, 0.024)	-0.012 (-0.048, 0.024)
Log-likelihood ratio test	0.059	0.273	0.017	0.268

Outcome: β (95%CI) *P*-value; β: regression coefficient, CI Confidence interval; Result variable: FT3/FT4, TT4RI, TSHI, TFQI; Exposed variable: CAP; Adjusted for gender, age, hypertension, diabetes, hyperlipidemia, FBG, HbA1c, TG, T-CHO, LDL-C, HDL-C, ALT, AST, GGT, TBIL, ALB, SCr, SUA.

### 3.10 The trend tests of multiple regression equations for the relationship between CAP and TH sensitivity in BMI subgroups

The results for BMI <28 kg/m² with CAP <277 dB/m (N = 9,312) of trend analysis of multiple regression equations between CAP and FT3/FT4 showed that as the CAP quartiles increased, the trend of gradually increased FT3/FT4 reached statistical significance; for each increased quartile in CAP, FT3/FT4 increased by 0.002 (β = 0.002, p < 0.00001).

**Figure 10:**
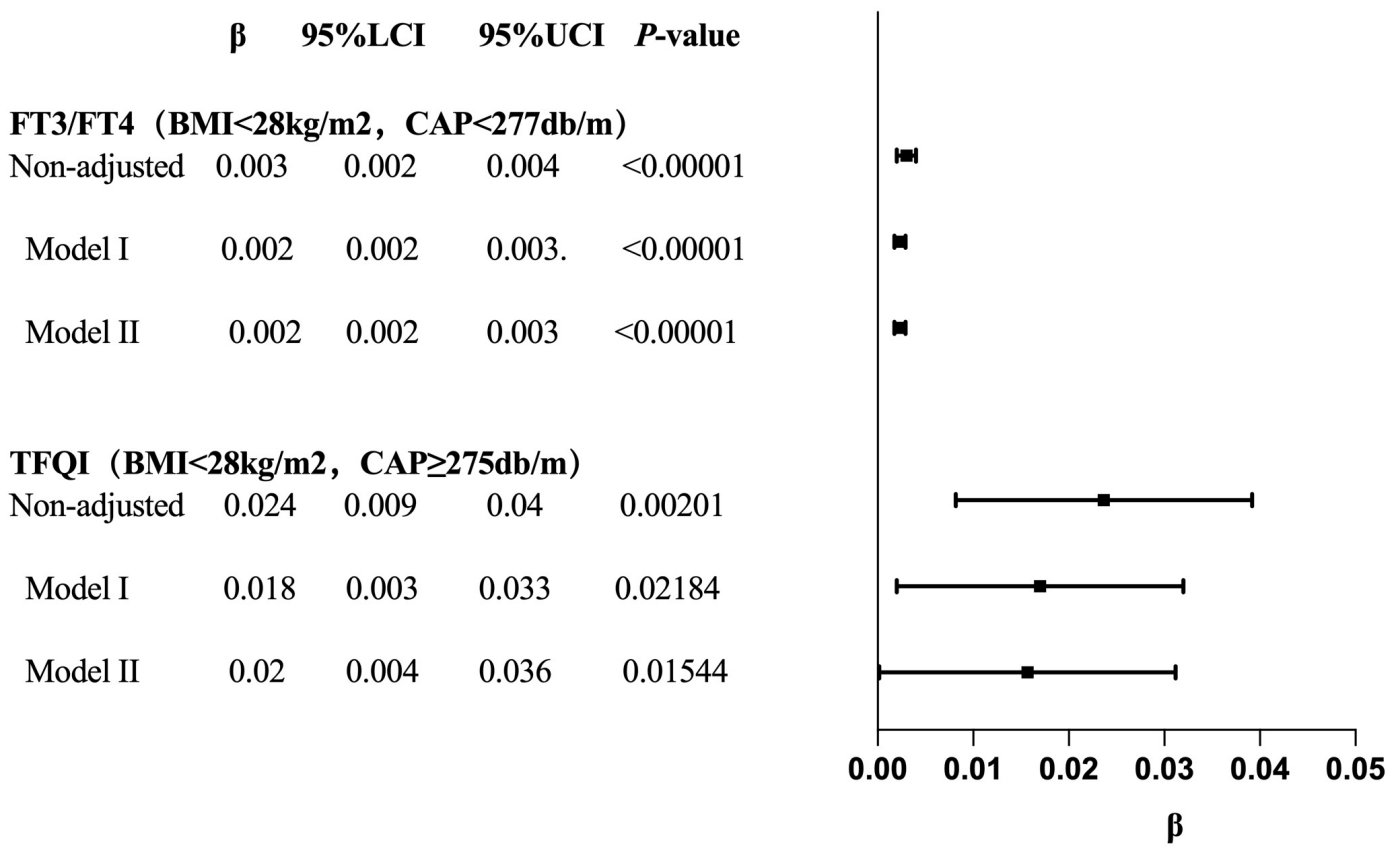
Forest map for the trend test results of CAP and TH sensitivity in BMI subgroup.

The results for BMI <28 kg/m² group with CAP ≥275 dB/m (N = 1,823) of trend analysis of multiple regression equations between CAP and TFQI showed that as the CAP quartiles increased, the trend of gradually increased TFQI reached statistical significance; for each increased quartile in CAP, TFQI increased by 0.016 (β = 0.016, p = 0.04805) (**Table 9**, **Figure 10**).

**Table 9:** Trend analysis of multiple regression equations of the relationship between CAP and TH sensitivity in BMI subgroups.

Exposure	Non-adjusted	*P*-value	Model I	*P*-value	Model II	*P*-value
	β (95%CI)		β (95%CI)		β (95%CI)	
FT3/FT4(BMI<28kg/m^2^, CAP<277db/m)						
CAP	0.000 (0.000, 0.000)	<0.00001	0.000 (0.000, 0.000)	<0.00001	0.000 (0.000, 0.000)	<0.00001
CAP quartile						
Q1	0		0		0	
Q2	0.003 (0.001, 0.006)	0.00677	0.003 (0.000, 0.005)	0.02658	0.003 (0.000, 0.005)	0.0227
Q3	0.008 (0.005, 0.010)	<0.00001	0.007 (0.004, 0.009)	<0.00001	0.007 (0.004, 0.009)	<0.00001
Q4	0.009 (0.006, 0.011)	<0.00001	0.007 (0.005, 0.009)	<0.00001	0.007 (0.004, 0.009)	<0.00001
CAP quartile continuous	0.003 (0.002, 0.004)		0.002 (0.002, 0.003)		0.002 (0.002, 0.003)	
*P* for trend	<0.00001		<0.00001		<0.00001	
TFQI(BMI<28kg/m^2^, CAP≥275db/m)						
CAP	0.002 (0.001, 0.004)	0.0012	0.002 (0.000, 0.003)	0.01765	0.001 (-0.000, 0.003)	0.0514
CAP quartile						
Q1	0		0		0	
Q2	0.048 (-0.001, 0.098)	0.05445	0.040 (-0.008, 0.088)	0.10105	0.044 (-0.003, 0.092)	0.069
Q3	0.051 (0.001, 0.102)	0.04716	0.046 (-0.003, 0.096)	0.06771	0.044 (-0.005, 0.093)	0.0794
Q4	0.080 (0.031, 0.130)	0.00143	0.058 (0.009, 0.106)	0.01998	0.050 (0.001, 0.099)	0.04618
CAP quartile continuous	0.024 (0.008, 0.039)		0.017 (0.002, 0.032)		0.016 (0.000, 0.031)	
*P* for trend	0.00257		0.02694		0.04805	

Outcome: β (95%CI) *P*-value, β: regression coefficient, CI Confidence interval; Result variable: FT3/FT4, TFQI; Exposed variable: CAP; Model I: Adjusted for gender, age; Model II: Adjusted for gender, age, hypertension, diabetes, hyperlipidemia, FBG, HbA1c, TG, T-CHO, LDL-C, HDL-C, ALT, AST, GGT, TBIL, ALB, SCr, SUA.

## 4 Discussion

Under physiological conditions, THs regulate the metabolism of triglycerides and cholesterol, increasing the activity of hepatic lipase and enhancing the mobilization of lipids within lipid droplets. The effects of THs on liver lipid homeostasis are mostly achieved through the transcriptional regulation of target genes involved in these homeostasis pathways (16). THs stimulate the breakdown of fat in white adipose tissue, generating circulating free fatty acids (FFAs), which are the main source of lipids for the liver (17). FFAs enter liver cells through fatty acid transport proteins, liver fatty acid–binding proteins, and fatty acid translocase (18). However, the specific mechanism by which THs regulate the hepatic uptake of FFAs remains unclear. As the core site for TH metabolism and transport, the liver produces the main TH transporters and regulates circulating THs. In turn, THs regulate the metabolism of liver cells and the production of bilirubin by regulating lipid metabolism (19). Therefore, THs and liver metabolism interact with and synergistically promote each other.

Much evidence indicates that the TH/THR axis is involved in the occurrence of MASLD. Abnormalities in the synthesis and secretion of THs are closely related to MASLD. Studies have shown that the incidence of MASLD in patients with hyperthyroidism was 11.97%, and the level of FT3 was negatively correlated with liver fat content in this population (20). For individuals with normal thyroid function, TSH was considered a risk factor for MASLD, and was associated with obesity, atherosclerotic dyslipidemia, metabolic syndrome (MetS), elevated transaminase levels, and changes in cholesterol and triglyceride levels (21). Moreover, some studies have reported that the levels of FT3 and FT4 were not significantly correlated with MASLD (22). However, other studies have shown that in the elderly population, high levels of FT3 within the normal range and low levels of TSH within the normal range could independently predict the incidence of MASLD (23). Thus, there are significant differences in many conclusions regarding the correlation between THs and MASLD.

In the early stage of hypothyroidism, the increase in liver steatosis was considered to be caused by decreased THs. Later, it was found that in addition to the harmful effect of decreased THs on the lipid homeostasis in the liver, an increase in TSH itself might promote the development of MASLD by stimulating hepatic fatogenesis. TSH binds to the TSH receptor in hepatocytes, promoting the expression of the rate-limiting enzyme 3-hydroxy-3-methylglutaryl-CoA reductase (HMGCR), which is involved in cholesterol synthesis. Exogenous TSH increased the expression of HMGCR in the liver and promoted cholesterol synthesis in hypothyroid rats with hypothyroidism (24). In rodents, TSHR was expressed in hepatocytes and was stimulated by TSH, which induced hepatic steatosis through SREBP1C (25). TSH inhibited the synthesis of bile acids in the liver through the SREBP2–HNF4α–CYP7A1 signaling pathway (26). It could also inhibit cholesterol synthesis by increasing AMPK-mediated phosphorylation of HMGCR and thereby inhibiting HMGCR activity (27). Overall, these findings support the view that TSH itself can regulate lipid and cholesterol homeostasis in the liver. However, the *in vivo* studies have shown that the direct effect of TSH, independent of thyroid hormones, is extremely difficult to explain, because when TSH levels increase, serum thyroid hormone levels usually decrease accordingly.

Our research showed that as the degree of MASLD fatty infiltration increased, the levels of FT3, FT4, and FT3/FT4 rose, while the level of TSH did not decrease accordingly. In the population with BMI <28 kg/m², low CAP levels were positively correlated with FT3/FT4, while high CAP levels were positively correlated with TFQI. In males, high CAP levels were negatively correlated with FT3/FT4 and positively correlated with TSHI and TFQI. Possible mechanisms include: 1. High levels of THs stimulate increased activity of type 1 deiodinase (DIO1) in the liver, leading to increased FT3/FT4 levels. This manifestation is especially prominent in the early stage of liver fatty infiltration and may represent a compensatory response exhibited by the body to promote hepatic fat metabolism in liver. 2. Moderate-to-severe fatty infiltration leads to reduced expression of TH receptors in hepatocytes and decreased peripheral TH sensitivity. 3. The expression of type 2 deiodinase (DIO2) in adipose tissue of overweight individuals is lower than that of normal-weight individuals, particularly in visceral fat, which is lower than that in subcutaneous fat tissue (28). It is speculated that the DIO2 is mainly distributed in the hypothalamic–pituitary axis, and the insufficient conversion of T3 in this tissue leads to inappropriate elevation or insufficient suppression of TSH or inability to decrease accordingly, resulting in reduced central TH sensitivity.

Our research suggests that the fat infiltration in MASLD may affect TH metabolism of THs and TH receptor expression of TH receptors, leading to TH resistance in the liver, and further influencing lipid metabolism and its regulation of the inflammatory regulation in MASLD. This may provide a theoretical basis for developing treatment strategies to improve MASLD by regulating TH metabolism and TH receptors, but further in-depth basic experiments are needed for verification.

## 5 Limitations

Firstly, the clinical data were analyzed from a single-center physical examination, which does not represent the characteristics of the general population. Secondly, although we had adjusted many confounding factors were adjusted for in the analysis, there may still have been unmeasured factors, such as incomplete medical history reports and unclear medication histories, which could affect the statistical results. Thirdly, data on thyroid peroxidase antibodies, thyroglobulin antibodies, and other related markers were not available. Therefore, the influence of thyroid diseases such as Hashimoto’s thyroiditis could not be ruled out.

## 6 Conclusion

TH sensitivity in patients with MASLD was significantly impaired. In non-obese individuals, mild liver fatty infiltration was associated with higher peripheral TH conversion rate and sensitivity, while moderate-to-severe fatty infiltration was related to lower central TH sensitivity. In males, severe liver fatty infiltration was associated with reduced peripheral and central TH sensitivity. The possible mechanism may be that hepatic fat deposition leads to abnormal expression of TH receptors; however, the specific mechanism requires further study.

## Data Availability

The raw data supporting the conclusions of this article will be made available by the authors, without undue reservation.

## References

[1] Rinella ME Lazarus JV Ratziu V Francque SM Sanyal AJ Kanwal F. A multisociety Delphi consensus statement on new fatty liver disease nomenclature. J Hepatol. (2023) 79:1542–56. doi: 10.1016/j.jhep.2023.06.003, PMID: 37364790

[2] Younossi ZM Golabi P Paik JM Henry A Van Dongen C Henry L. The global epidemiology of nonalcoholic fatty liver disease (NAFLD) and nonalcoholic steatohepatitis (NASH): a systematic review. Hepatol (Baltimore Md). (2023) 77:1335–47. doi: 10.1097/HEP.0000000000000004, PMID: 36626630 PMC10026948

[3] Younossi ZM. Non-alcoholic fatty liver disease-A global public health perspective. J Hepatol. (2019) 70:531–44. doi: 10.1016/j.jhep.2018.10.033, PMID: 30414863

[4] Veracruz N Hameed B Saab S Wong RJ. The association between nonalcoholic fatty liver disease and risk of cardiovascular disease, stroke, and extrahepatic cancers. J Clin Exp Hepatol. (2021) 11:45–81. doi: 10.1016/j.jceh.2020.04.018, PMID: 33679048 PMC7897860

[5] Biondi B Kahaly GJ Robertson RP. Thyroid dysfunction and diabetes mellitus: two closely associated disorders. Endocrine Rev. (2019) 40:789–824. doi: 10.1210/er.2018-00163, PMID: 30649221 PMC6507635

[6] Gavrila A Hollenberg AN. The hypothalamic-pituitary-thyroid axis: physiological regulation and clinical implications. Thyroid Its Dis. (2019), 13–23. doi: 10.1007/978-3-319-72102-6_2

[7] Laclaustra M Moreno-Franco B Lou-Bonafonte JM Mateo-Gallego R Casasnovas JA Guallar-Castillon P. Impaired sensitivity to thyroid hormones is associated with diabetes and metabolic syndrome. Diabetes Care. (2019) 42:303–10. doi: 10.2337/dc18-1410, PMID: 30552134

[8] Jostel A Ryder WD Shalet SM. The use of thyroid function tests in the diagnosis of hypopituitarism: definition and evaluation of the TSH Index. Clin Endocrinol. (2009) 71:529–34. doi: 10.1111/j.1365-2265.2009.03534.x, PMID: 19226261

[9] Yagi H Pohlenz J Hayashi Y Sakurai A Refetoff S. Resistance to thyroid hormone caused by two mutant thyroid hormone receptors beta, R243Q and R243W, with marked impairment of function that cannot be explained by altered *in vitro* 3,5,3’-triiodothyroinine binding affinity. J Clin Endocrinol Metab. (1997) 82:1608–14. doi: 10.1210/jc.82.5.1608, PMID: 9141558

[10] Wu Z Jiang Y Li P Wang Y Zhang H Li Z. Association of impaired sensitivity to thyroid hormones with hyperuricemia through obesity in the euthyroid population. J Trans Med. (2023) 21:436. doi: 10.1186/s12967-023-04276-3, PMID: 37403157 PMC10320931

[11] Ding X Wang Y Liu J Wang G. Impaired sensitivity to thyroid hormones is associated with elevated homocysteine levels in the euthyroid population. J Clin Endocrinol Metab. (2022) 107:e3731–e7. doi: 10.1210/clinem/dgac371, PMID: 35708733

[12] Nie X Ma X Xu Y Shen Y Wang Y Bao Y. Increased serum adipocyte fatty acid-binding protein levels are associated with decreased sensitivity to thyroid hormones in the euthyroid population. Thyroid: Off J Am Thyroid Assoc. (2020) 30:1718–23. doi: 10.1089/thy.2020.0011, PMID: 32394790

[13] Liu B Wang Z Fu J Guan H Lyu Z Wang W. Sensitivity to thyroid hormones and risk of prediabetes: A cross-sectional study. Front Endocrinol. (2021) 12:657114. doi: 10.3389/fendo.2021.657114, PMID: 34017311 PMC8129566

[14] Zhang X Chen Y Ye H Luo Z Li J Chen Z. Correlation between thyroid function, sensitivity to thyroid hormones and metabolic dysfunction-associated fatty liver disease in euthyroid subjects with newly diagnosed type 2 diabetes. Endocrine. (2023) 80:366–79. doi: 10.1007/s12020-022-03279-2, PMID: 36539681

[15] Knapp TR. Statistical power analysis for the behavioral-sciences, 2nd edition - Cohen, J. Educ psychol Measurement. (1990) 50:225–7. doi: 10.1177/0013164490501028

[16] Singh BK Sinha RA Zhou J Tripathi M Ohba K Wang ME. Hepatic FOXO1 target genes are co-regulated by thyroid hormone via RICTOR protein deacetylation and MTORC2-AKT protein inhibition. J Biol Chem. (2016) 291:198–214. doi: 10.1074/jbc.M115.668673, PMID: 26453307 PMC4697156

[17] Sinha RA Singh BK Yen PM. Direct effects of thyroid hormones on hepatic lipid metabolism. Nat Rev Endocrinol. (2018) 14:259–69. doi: 10.1038/nrendo.2018.10, PMID: 29472712 PMC6013028

[18] Mashek DG. Hepatic fatty acid trafficking: multiple forks in the road. Adv Nutr (Bethesda Md). (2013) 4:697–710. doi: 10.3945/an.113.004648, PMID: 24228201 PMC3823518

[19] Piantanida E Ippolito S Gallo D Masiello E Premoli P Cusini C. The interplay between thyroid and liver: implications for clinical practice. J Endocrinol Invest. (2020) 43:885–99. doi: 10.1007/s40618-020-01208-6, PMID: 32166702

[20] Wang B Wang B Yang Y Xu J Hong M Xia M. Thyroid function and non-alcoholic fatty liver disease in hyperthyroidism patients. BMC Endocrine Disord. (2021) 21:27. doi: 10.1186/s12902-021-00694-w, PMID: 33602203 PMC7890885

[21] Martínez-Escudé A Pera G Costa-Garrido A Rodríguez L Arteaga I Expósito-Martínez C. TSH levels as an independent risk factor for NAFLD and liver fibrosis in the general population. J Clin Med. (2021) 10:2907. doi: 10.3390/jcm10132907, PMID: 34209831 PMC8267939

[22] Tan Y Tang X Mu P Yang Y Li M Nie Y. High-normal serum thyrotropin levels increased the risk of non-alcoholic fatty liver disease in euthyroid subjects with type 2 diabetes. Diabetes Metab Syndrome Obes: Targets Ther. (2021) 14:2841–9. doi: 10.2147/DMSO.S313224, PMID: 34188507 PMC8235944

[23] Gu Y Wu X Zhang Q Liu L Meng G Wu H. High-normal thyroid function predicts incident nonalcoholic fatty liver disease among middle-aged and older euthyroid subjects. J Gerontol Ser A Biol Sci Med Sci. (2022) 77:197–203. doi: 10.1093/gerona/glab037, PMID: 33534875

[24] Tian L Song Y Xing M Zhang W Ning G Li X. A novel role for thyroid-stimulating hormone: up-regulation of hepatic 3-hydroxy-3-methyl-glutaryl-coenzyme A reductase expression through the cyclic adenosine monophosphate/protein kinase A/cyclic adenosine monophosphate-responsive element binding protein pathway. Hepatol (Baltimore Md). (2010) 52:1401–9. doi: 10.1067/mem.2000.105659, PMID: 20648556

[25] Yan F Wang Q Lu M Chen W Song Y Jing F. Thyrotropin increases hepatic triglyceride content through upregulation of SREBP-1c activity. J Hepatol. (2014) 61:1358–64. doi: 10.1016/j.jhep.2014.06.037, PMID: 25016220

[26] Song Y Xu C Shao S Liu J Xing W Xu J. Thyroid-stimulating hormone regulates hepatic bile acid homeostasis via SREBP-2/HNF-4α/CYP7A1 axis. J Hepatol. (2015) 62:1171–9. doi: 10.1016/j.jhep.2014.12.006, PMID: 25533663

[27] Zhang X Song Y Feng M Zhou X Lu Y Gao L. Thyroid-stimulating hormone decreases HMG-CoA reductase phosphorylation via AMP-activated protein kinase in the liver. J Lipid Res. (2015) 56:963–71. doi: 10.1194/jlr.M047654, PMID: 25713102 PMC4409286

[28] Kurylowicz A Jonas M Lisik W Jonas M Wicik ZA Wierzbicki Z. Obesity is associated with a decrease in expression but not with the hypermethylation of thermogenesis-related genes in adipose tissues. J Trans Med. (2015) 13:31. doi: 10.1186/s12967-015-0395-2, PMID: 25622596 PMC4314800

